# Supplementary figures and images for: BES1/BZR1 Family Transcription Factors Regulate Plant Development via Brassinosteroid-Dependent and Independent Pathways
Source: Int J Mol Sci. 2022 Sep 5;23(17):10149. doi: 10.3390/ijms231710149 (PMC9478962; doi:10.3390/ijms231710149)

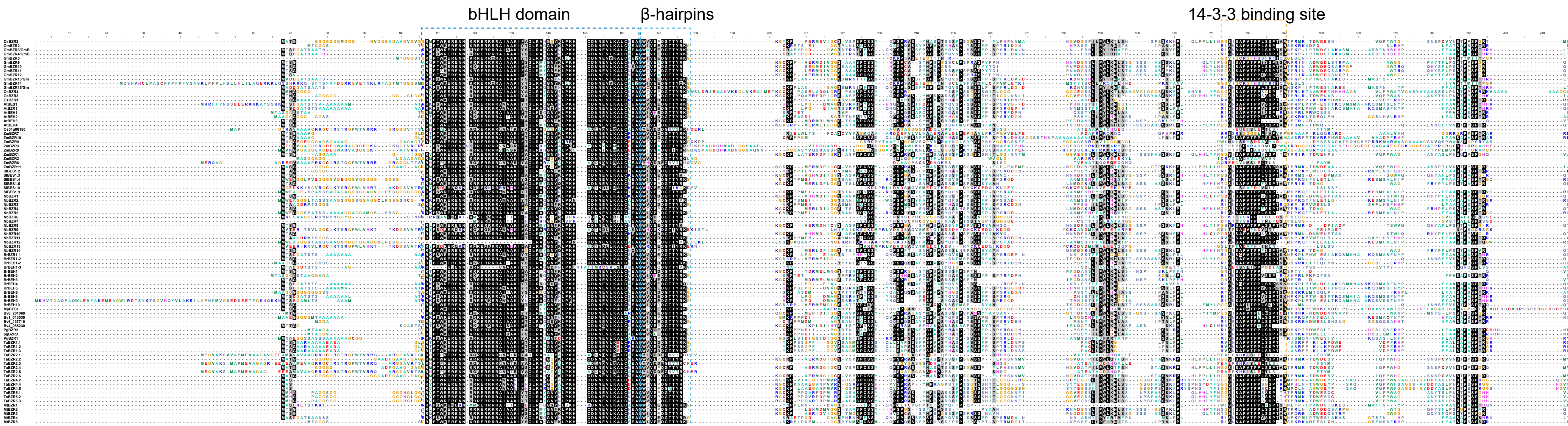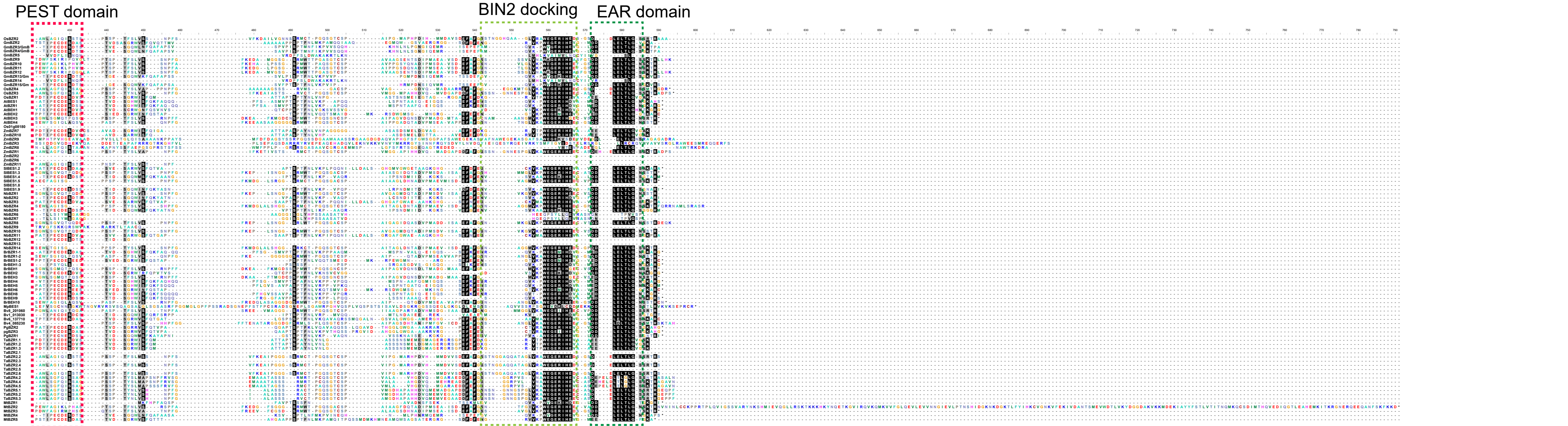

Supplement: Supplementary file 1 [file ijms-23-10149-s001.zip › Supplementary Figure S1 The result of Clustal Omega.pdf]
